# Arbuscular mycorrhizal fungal community composition determines the competitive response of two grassland forbs

**DOI:** 10.1371/journal.pone.0219527

**Published:** 2019-07-10

**Authors:** Lena Neuenkamp, Martin Zobel, Eva Lind, Maret Gerz, Mari Moora

**Affiliations:** 1 Institute of Ecology and Earth Sciences, University of Tartu, Tartu, Estonia; 2 Institute of Plant Sciences, University of Bern, Bern, Switzerland; University of California Berkeley, UNITED STATES

## Abstract

We performed a greenhouse experiment to assess how differences in AM fungal community composition affect competitive response of grassland plant species. We used a full factorial design to determine how inoculation with natural AM fungal communities from different habitats in Western Estonia affects the growth response of two grassland forbs (*Leontodon hispidus* L., *Plantago lanceolata* L.) to competition with a dominant grass (*Festuca rubra* L.). We used AM fungal inocula that were known to differ in AM fungal diversity and composition: more diverse AM fungal communities from open grasslands and less diverse AM fungal communities from former grassland densely overgrown by pines (young pine forest). The presence of AM fungi balanced competition between forb and grass species, by enhancing competitive response of the forbs. The magnitude of this effect was dependent on forb species identity and on the origin of the AM fungal inoculum in the soil. The grassland inoculum enhanced the competitive response of the forb species more effectively than the forest inoculum, but inoculum-specific competitive responses varied according to the habitat preference of the forb species. Our findings provide evidence that composition and diversity of natural AM fungal communities, as well as co-adaptation of plant hosts and AM-fungal communities to local habitat conditions, can determine plant-plant interactions and thus ultimately influence plant community structure in nature.

## Introduction

Understanding the factors that determine plant community structure is a key aim of ecology. Competition is believed to be a fundamental process determining plant species coexistence and the structure of plant communities [1–3]. Several abiotic and biotic factors influence competition between plants, including climate and nutrient availability but also interactions with other organisms including pathogens, herbivores and mutualists [4–6]. The role of microbial interactions, such as the symbiosis between plants and arbuscular mycorrhizal (AM) fungi, has received increasing attention in empirical and conceptual studies investigating plant competition during the recent decades [4, 7–9].

AM fungi are a group of obligatory endophytes (phylum Mucoromycota, subphylum Glomeromycotina, [10]) that form symbiosis with the majority of land plants. These fungi can improve plant nutrient uptake [11], alleviate plant abiotic stress [12, 13] and increase plant resistance to pathogens [14] in exchange for plant-assimilated carbon. Through these mechanisms, AM fungi can influence plant performance and, since the effects on plant performance vary in relation to the plant and AM fungal species involved [15–17], they can also mediate plant coexistence. There is experimental evidence that AM fungi mediate plant coexistence by providing asymmetric benefits to competing plant species and thus altering competitive hierarchies (e.g. [18–23], and see [24] for a review on that topic). AM fungi often confer a competitive advantage to the more mycotrophic plant species e.g. [18, 21], and higher AM fungal diversity or the presence of particularly beneficial AM fungal taxa can enhance this advantage [16, 19, 22, 25].

A shortcoming of most of earlier experiments is that the fungal inocula used contained only a low number AM fungal taxa, and predominantly consisted of commercially cultured AM fungi [17, 26]. In nature, plant individuals are simultaneously colonised by several AM fungal taxa [27, 28] and culturable taxa may be a functionally distinct subset of all AM fungal taxa [29]. Moreover, unculturable taxa appear to be more abundant than culturable taxa among AM fungal communities in natural ecosystems [29, 30]. These characteristics considerably limit the scope of inferences that can be drawn from many published experiments. They may be most relevant to early successional ecosystems: conditions where AM fungal populations are dispersal limited, local AM fungal communities are species-poor and dominated by culturable AM fungal taxa cf. [30]. In this case, the lack or low abundance of suitable AM fungal partners might impose a disadvantage to mycotrophic over non-/weakly mycotrophic plant species (`coarse scale effects´ sensu [4, 7]). Experiments using natural AM fungal inoculum would allow inferences to be drawn about successionally mature ecosystems, where AM fungi are abundant, and thus `fine scale effects´ (sensu [4]) of AM fungi are relevant for plant interactions. In such systems, AM fungal community composition, multifunctionality and shared mycelial networks are likely to influence plant-plant interactions [7]. This kind of competition experiment is rare (but see[23, 31]) although differential effects of natural AM fungal communities on plants grown singly [32–35] suggest that variation in AM fungal community composition may influence plant co-existence in nature.

Indeed, [36] recorded correlation of plant and AM fungal communities along a successional gradient from open dry grassland towards forest, indicating that community assembly of both symbionts was linked in that system. The authors proposed AM-fungal mediated plant competition as one potential mechanism linking both communities. In particular, if AM fungi enhance competitive response of a plant species, they may equalize interspecific competition and hence enhance plant coexistence (cf. [37]). The strength of correlation found by [36] varied in relation to habitat characteristics—strongest correlation was observed in open grassland stands, with weaker correlation in young pine forest stands. These findings suggest that the degree to which AM fungi mediate plant-plant competition and plant coexistence may be habitat specific, depending on the composition of the local AM fungal and the plant community. One may hypothesize that the link between plant and AM fungal communities was stronger in grasslands compared to forests due to a stronger effect of the more diverse grassland AM fungal community on plant competitive response (sensu [38] and [39]), balancing competitive hierarchies, thus preventing competitive exclusion, and ultimately contributing to the high plant diversity in grasslands. Moreover, co-adaptation of local plant and AM fungal communities could be an additional factor influencing plant responses to mycorrhizal fungi [40, 41]. Thus, mycorrhizal effects on plant competition will likely also depend on the origin of the plant species and AM fungal communities involved–with stronger effects in parings of plants and AM fungi from the same habitat.

We performed a greenhouse experiment to test these ideas and assess how differences in AM fungal community composition affect competitive response (sensu [38]) of two focal grassland plant species. We designed an additive competition experiment (sensu [42]) to determine the effect of inoculation with natural AM fungal communities from different habitats in Western Estonia on the growth response of two focal grassland forbs (*Leontodon hispidus* L., *Plantago lanceolata* L.) to competition with an associate grass species (*Festuca rubra* L.). We further tested whether mycorrhizal effects on competitive response depended on the identity of the focal plant and the origin of the AM fungal inoculum (open grassland and former grassland densely overgrown by pines i.e. young pine forest). The inocula were stemming from the field sites studied in [36] from which we knew that AM fungal diversity, composition and abundance, as well as plant-AM fungal relationships differed between both habitat types. Grassland soils harboured a higher diversity and abundance of AM fungi as well as exhibited a stronger correlation between plants and AM fungal composition than forest soils [36]. All experimental plant species are AM-forming native grassland species, but differ in their reliance on mycorrhizal symbiosis and their habitat preference at the study site. The focal species *P*. *lanceolata* occurred only in grasslands, *L*. *hispidus* was present in grasslands, but occurred predominantly in young forest, and the associate species *F*. *rubra* was equally abundant in both habitat types [36]. *Festuca rubra* can be expected to rely less on mycorrhizal symbiosis than the forb species [17, 36], due to efficient nutrient-uptake with a fine, well-branched root system, which is typical of C3-grasses [43, 44]. We estimated focal plant growth responses to competition using the relative interaction index (RII, [45]) with an increase in the index value indicating a stronger competitive response of the focal plant i.e. less growth suppression due to competition (sensu [39]). We hypothesized that:

(H1) The presence of AM fungal inoculum from either community will increase the competitive response i.e. will reduce biomass loss in competition of both focal species due to larger benefits of inoculation to the more mycotrophic focal forb species compared to the less mycotrophic associate grass species.
(H2) The magnitude of AM fungal effects on competitive responses of the focal species will depend on the origin of the AM fungal inoculum: effects will be larger in the presence of grassland compared with young forest inoculum due to higher AM fungal diversity and abundance in the former than the latter.
(H3) The magnitude of AM fungal effects on competitive responses of focal species will depend on the correspondence of inoculum origin and the habitat preference of the focal species: competitive response in the presence of the grassland inoculum will be greater for *P*. *lanceolata* than for *L*. *hispidus*, and *vice versa* in the presence of the forest inoculum.

## Material and methods

### Soil inoculum

We chose dry calcareous species-rich grasslands as a model system for our experiment. These grasslands developed historically under the impact of long-term extensive grazing by domestic animals, mowing for hay and the cutting of shrubs and trees for fuel. They traditionally had a maximum juniper (*Juniperus communis*) cover of 30%, but are now increasingly overgrown with Scots pine (*Pinus sylvestris*; up to 80% cover) due to a gradual decline in historical land-use practices since the 1980s [46].

We used whole AM fungal communities present in the soil of open grasslands and adjacent young forests all situated within a study site of 2ha (Western Estonia; 58.62N, 23.54E) as natural inocula for our experiment. The study site is public thus requiring no extra permission for the accession of the site and the collection of the experimental soil used as inoculum. The grassland stands form a temporal sequence of regeneration succession following cessation of management, characterised by gradual shrub and tree encroachment (see [36] for details; Fig A in S2 File): from continuously managed (mown) open grassland (grassland soil) towards young pine forests (forest soil) where management ceased about 60 years ago. We chose these inocula as molecular analyses by [36] showed that plant and AM fungal richness and composition significantly differed between open grasslands and young pine forests at the same study site (Table 1). In April 2014, we collected topsoil (3–10 cm) from ten randomly chosen locations in both stands. Soil was sieved to remove roots with a fine-mesh kitchen sieve, pooled per stand and carefully mixed.

**Table 1:** Compositional characteristics of the source habitats of the inocula. Shown are compositional characteristics (mean ±SE) of grassland and young forest stands based on data collected from the same study site earlier (see [36] for details). VT: virtual taxa, i.e. phylogenetically defined sequence groups roughly corresponding to species-level taxa, cf. [47]. Fatty acid marker NLFA 16w:5 is a reliable marker to estimate AM fungal abundance [48].

Stand	species richness (species number per sampling unit; plants: 1x1m, AM fungi: 0.1x0.1x0.1m)		species abundance (plants: cover per 1x1m, AM fungi: fatty acid marker NLFA 16w:5 (nmol) per g soil)			
	Plants	AM fungi	*Leontodon hispidus*	*Plantago lanceolata*	*Festuca rubra*	AM fungi
open grassland	31.7±0.7	30.4±1.2	0.2±0.1	0.9±0.2	1.5±0.2	5.9 ± 0.9
young pine forest	21.2±1.2	20.7±1.3	4.1±0.7	0.1±0.1	1.2±0.4	3.9 ± 0.6

We produced two kinds of inocula (grassland inoculum, forest inoculum) and one control. Inocula were prepared as 1:1:1 mixtures of live and/or sterilized (gamma radiation, 25 kGy, Scandinavian Clinics Estonia OÜ, Harjumaa) grassland soil, forest soil and autoclaved sand (121°C, 60 min), in order to maintain constant soil chemical properties among inocula. For the grassland inoculum, the grassland soil was live and other components sterilized or autoclaved; for the forest inoculum, only the forest soil was live; while the non-mycorrhizal control inoculum contained only sterilized soils and autoclaved sand.

The whole soil inocula (grassland and forest inoculum) that we used in the greenhouse experiment are expected to contain the complete biotic soil community i.e. organisms including but not exclusive to mycorrhizal fungi. Yet, inoculation effects stemming from whole soil inocula can be related to AM fungal effects, if they are compared to a control, which has received a microbial filtrate (wash) containing the majority of soil organisms of the experimental soil, except AM fungi (pore size 50 μm) [49]. This method works effectively for AM fungi due to their relatively large spores compared to the majority of other soil microbial communities (e.g. bacteria and non-AM fungi) [35, 50]. Thus, all pots–containing live inocula and control treatments—received 40 ml of filtered microbial wash from mixed grassland and forest soil inocula to correct for possible differences in the soil bacterial and non-AM fungal communities (pore size 50 μm).

Average soil nutrient levels of the pots can influence plant growth response to AM fungal inoculation and thereby the growth of AM fungi [17, 50]. All pots contained equal amounts of grassland soil, forest soil and sand. Thus, soil geochemical properties of soil mixtures used as inocula can be expected be equal among pots and to reflect the average properties of both natural soils used for inoculation or even slightly lower due to the addition of one third of nutrient-poor sand to all pots. We assessed the soil geochemical properties in the soils used for inoculation in the field during our previous field study [36]. Both soils used for inoculation were rather low in available phosphorus (P; both inocula: P = 0.02±g 0.00 per kg soil) and total nitrogen (N), with the grassland soil being poorer in nitrogen than the forest soil (grassland soil: N_tot_ = 3.98±0.26g per kg soil; forest soil: N_tot_ = 7.01±0.56g per kg soil), yet they were rich in calcium (Ca; grassland soil: Ca = 2.86±0.10g per kg soil; forest soil. Ca = 3.98±0.15g per kg soil). Grassland soils were poorer in the amount of organic carbon per kg soil (C_org_) compared to forest soils (grassland soil: C_org_ = 45.54±2.28 g per kg soil; forest soil: C_org_ = 80.78±6.01 g per kg soil). See S3 Table in [36] for details on soil geochemical characteristics of the field soils from where we collected the soil inocula used in this experiment.

### Greenhouse experiment

We used the additive experimental design suggested by [38, 39] to measure the response of the focal taxa to competition by an associate taxon [42]. The ranking of competitive response of the focal taxa should be determined by their ability to tolerate depleted resource levels in the presence of the associate taxon [38]. We used plant growth (aboveground and belowground biomass) as response parameter to estimate competitive response.

In particular, we addressed the effect of AM fungal inoculum origin (different successional stages of calcareous grassland) on the competitive response of two forb species (*Leontodon hispidus*, L., *Plantago lanceolata*, L.; i.e. focal species) an associate grass species (*Festuca rubra*, L). All experimental plant species were native to the study site, but showed different distribution/habitat preferences among the grassland and forest stands (Table 1) [36]. *Festuca rubra* was equally distributed in grassland and forest stands, *L*. *hispidus* was more abundant in forest stands and *P*. *lanceolata* more abundant in grassland stands. For *P*. *lanceolata* and *L*. *hispidus*, we used seeds collected during summer 2013 from local grassland stands in the region of the study site. For *F*. *rubra*, we used seeds from a certified, local Estonian seed producer (type KAUNI, EE12-59663, C category). All seeds were sterilized in a 0.01% solution of potassium permanganate (KMnO_4_) and germinated in May 2014 for five weeks in autoclaved sand. Neither the micro-organisms (AM fungi) inhabiting the soil inocula nor the native plant seeds collected for the experiment were protected.

We performed our experiment at the greenhouse facilities of the Department of Botany, University in Tartu (58.34N, 26.72E). Five weeks after seedling emergence, we transplanted seedlings into experimental pots (diameter 10.5 cm, height 12 cm), one seedling of the focal forb species *L*. *hispidus* or *P*. *lanceolata* to the centre of each pot. We grew half of the focal seedlings alone (a single seedling per pot), and the other half in competition with the associate grass species *F*. *rubra*, i.e. surrounded by four *F*. *rubra* seedlings. All focal seedlings, single or competing, were grown with either living grassland or forest inoculum including AM fungi or with control soil lacking AM fungi. There were ten replicates for each combination of competition (single, competition) and soil (grassland, forest, non-mycorrhizal control) treatment, making 120 pots in total. In this experiment we explicitly focussed on the effect of inoculation on the competitive response of the two focal forb species and thus assessed growth response to inoculation of seedlings grown alone only for the focal forb species but not the associate grass species.

Plants were grown in pots for 15 weeks, and all pots were randomly placed on greenhouse benches under a 16h-day: 8h-night illumination cycle and watered regularly. To control for heterogeneity in light-availability within the greenhouse the position of pots was changed randomly every four weeks. During the first three weeks, we replaced dead seedlings with living ones. At the end of the experiment, we harvested all plants, separated root- and shoot-biomass of all plant species, dried them for 24h at 55°C and measured the dry-weight of total root and shoot biomass per plant individual (focal species) or for all individuals per plot (associated species).

### Root colonization

In order to assess whether inoculation was successful, percentage colonization by AM fungi of the roots of *L*. *hispidus* and *P*. *lanceolata* was estimated from five root subsamples for every treatment combination (n = 5x3x2 = 30 individual samples). We stained the roots with trypan blue [51], mounted them on microscope slides and estimated colonization using the magnified grid–line intersection method [52]. In each sample, presence and absence of AM structures (hyphae, vesicles, arbuscules, coils) were scored for at least 120 intersections of the root and the vertical crosshair using an Olympus CH20 microscope at 400 x magnification. An intersection was considered mycorrhizal if the vertical crosshair intersected any AM structure. We found no root colonization for *L*. *hispidus* and *P*. *lanceolata* roots that were growing in the non-mycorrhizal control, and thus present colonization results only for grassland and forest soil inocula.

### Statistical analyses

We used the relative interaction index (RII) [45] to calculate plant responses to competition and inoculation (Table 2):

$$RII=\frac{{biomass}_{treat}-mean({biomass}_{control})}{{biomass}_{treat}+mean({biomass}_{control})}$$ (Eq 1)

where biomass_treat_ represents biomass of plants exposed to an experimental treatment (e.g. presence of the associate species or addition of inoculum) and biomass_control_ represents biomass of plants in the absence of the treatment (e.g., absence of competitors or inoculum). Responses to competition (RIIc) and inoculation (RIIi) were calculated in this way for two focal forb species (*L*. *hispidus and P*. *lanceolata)* and for three biomass types (root, shoot, and total biomass). RIIi was additionally calculated from the perspective of the grass species used as associate species (*F*. *rubra*) in our experiment. RII is symmetrical around zero, bounded by -1 and 1 [45]. RIIc < 0 indicates a decrease in focal biomass when grown in mixture (i.e. competition or a weak competitive response of the focal species (sensu [38])); RIIc > 0 indicates an increase in focal biomass when grown in mixture (i.e. facilitation or a strong competitive response of the focal species (sensu [38])). RIIi < 0 indicates a decrease in focal biomass when grown with AM fungal inoculation (i.e. parasitism); RIIi > 0 indicates an increase in focal biomass when grown with AM fungal inoculation (i.e. mutualism) [53].

**Table 2:** Summary table of response variables, hypotheses and predicted relationships.

Response variables		Calculation
RIIc: focal plant competitive response to with *F*. *rubra*^1^		$RIIc=\frac{{biomass}_{mix}-mean({biomass}_{single})}{{biomass}_{mix}+mean({biomass}_{single})}$
RIIi: focal or associate growth response to inoculation^2^		$RIIi=\frac{{biomass}_{inoc}-mean({biomass}_{control})}{{biomass}_{inoc}+mean({biomass}_{control})}$
dRIIi: differences in plant growth benefits from inoculation for focal and associate species, when grown in competition with each other		*dRIIi* = *RIIi*_*focal*_−*RIIi*_*associate*_
Hypotheses		Expected relationships
H1a	positive AM fungal effects on competitive response of the focal forb species (i.e. reduction of biomass loss)	*RIIc*_*grassland*/*forest*_>*RIIc*_*control*_
H1b	more positive AM fungal effects on the growth of the more mycotrophic (focal) forb than the less mycotrophic (associate) grass species when grown in competition with each other	*dRIIi*>0, i.e. *RIIi*_*focal*_>*RIIi*_*associate*_
H2	more positive AM fungal effects on the competitive response of the focal forb species with the grassland compared with the young pine forest inoculum	*RIIc*_*grassland*_>*RIIc*_*forest*_
H3	for each inoculum, AM fungal effects on competitive response are most positive for the focal species preferring the habitat from where the respective inoculum originates (*P*. *lanceolata*—grassland; *L*. *hispidus*—young pine forest)	grassland: *RIIc*_*P*. *lan*._>*RIIc*_*L his*._ forest: *RIIc*_*P*. *lan*._<*RIIc*_*L his*._

^1^ biomass_mix_ denotes biomass values of focal plants grown with associate plants; biomass_single_ denotes biomass values of plants grown singly.

^2^ biomass_inoc_ denotes biomass values of plants grown in the presence of AM fungal inoculum; biomass_control_ denotes biomass values of plants grown in the absence of AM fungal inoculum

Inoculation effects on focal plant response to competition (RIIc) do not directly indicate a competitive benefit to the focal species over the associate species. This is because inoculation could also influence the biomass of the associate species *F*. *rubra*, although it is considered less responsive to AM fungi than the focal forbs. Thus, an increase in focal biomass in response to inoculation might reflect benefits from mycorrhizal inoculation to focal competitive response, but also mycorrhizal growth benefits to the associate. To assess the net effect of inoculation to the competitive response of the focal species we compared inoculation effects for focal and associate species, when grown in competition with each other. We calculated the difference in RIIi (dRIIi) for focal and associate species grown together in the same pot:

$$dRIIi={RIIi}_{focal}-{RIIi}_{associate}$$ (Eq 2)

dRII was calculated in this way for two focal species (*L*. *hispidus*, *P*. *lanceolata*) and three biomass types (root, shoot, total biomass). dRIIi < 0 indicates amplified competition due to inoculum-mediated weaker competitive response of the focal species i.e. a greater growth benefit of inoculation for the associate compared with the focal species, and dRIIi > 0 indicates reduced competition due to inoculum-mediated stronger competitive response of the focal species) i.e. a greater growth benefit of inoculation for the focal species compared to the associate species [7]. Extreme values of dRIIi, where dRIIi > 0, might indicate out-competition of the associate by the focal species. Such a consequence appears unlikely in this experimental setup, as four associate plant individuals were grown together with one focal species, and hence *F*. *rubra* is the dominant species in all pots, with associate plant biomass being always higher than the biomass of the subordinate focal species. Thus, dRIIi > 0 indicates that inoculation has reduced the biomass difference between focal and associate species by promoting focal biomass more than associate plant biomass and thereby balanced interspecific competition, cf. [7]. The translation of hypotheses H1-H3 into predicted relationships among these indices are summarised in Table 2. Linear models were used to model variation in the indices in relation to focal species identity, inoculum origin and their interaction.

Shifts in percentage root colonization of focal species in response to inoculation and competition were assessed in the same manner as for plant biomass. Percentage root colonization by different types of AM fungal structures (hyphae, vesicles, arbuscules, coils) and relative response of percentage root colonization to competition (RIIc) were calculated according to Eq (1) and were used as response variables in linear models; inoculum origin or the interaction term of inoculum origin and focal species were used as explanatory variables.

All statistical analyses were carried out using the *stats* package in R (ver. 3.1.0; R Core Team 2014) in the RSTUDIO environment (ver. 0.98.932). Analysed data on plant biomass, competitive response and growth response to inoculation, as well as plant root colonization by AM fungi can be found in Tables A–C in S1 File.

## Results

### AM fungal root colonization of focal plants

Investigation of plant root colonization revealed that inoculation resulted in abundant colonization of *P*. *lanceolata* and *L*. *hispidus* roots by AM fungal structures: most abundantly by fungal hyphae (on average 80% of root length) and arbuscules (on average 40% of root length); coils and vesicles were less abundant (on average 1–3% of root length) (Table C in S1 File). No AM fungal structures were detected in the roots of focal plants grown without AM fungal inoculum. AM fungal root colonization was higher for *L*. *hispidus* than *P*. *lanceolata* when focal plants were grown without *F*. *rubra*. Only coil colonization differed in response to the inoculum origin, with higher colonization for the grassland compared with the forest inoculum (Table A in S2 File). When plants were grown with *F*. *rubra*, only the degree of hyphal and vesical colonization changed in response to competition. Hyphal colonization was higher when focal plants were grown with *F*. *rubra* compared to when grown without *F*. *rubra*, while response of vesicular colonization to the presence of *F*. *rubra* varied in relation to the inoculum origin (Table B in S2 File) Competition with *F*. *rubra* led to a decrease in vesicular colonization when plants were grown in grassland inoculum (RIIc<0), but an increase when they were grown in forest inoculum (RIIc>0; Table E in S2 File).

### Inoculation effects on plant growth response to competition

**Fig 1:**
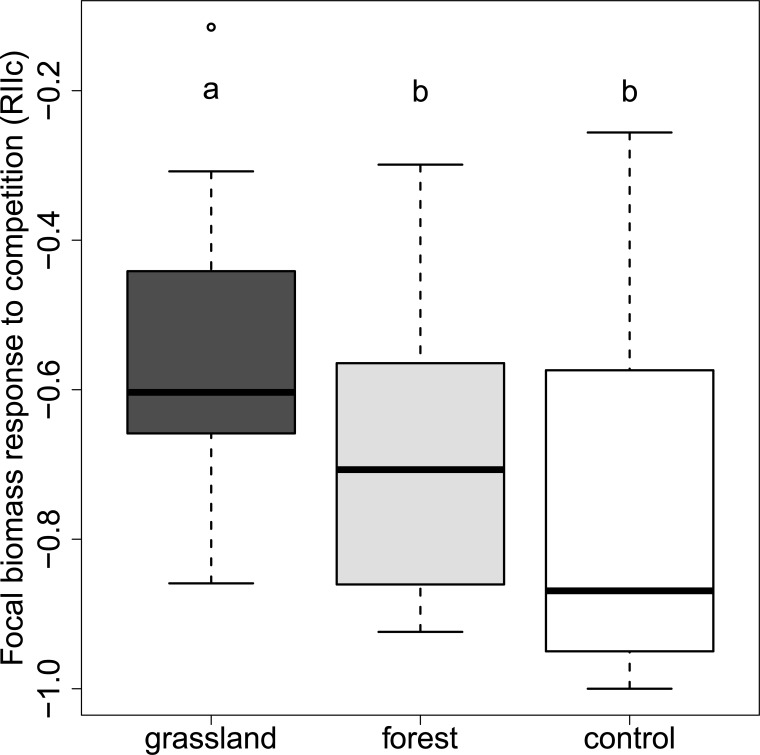
Competitive response of both focal species in relation to inoculum origin. The competitive response of both focal species was measured based on the index RII (RIIc, Eq (1)). Different letters indicate significant differences according to post hoc tests (p<0.05). Black lines indicate median values, boxes enclose quartiles, whiskers enclose 95% confidence intervals, and points indicate outlier values. grassland = grassland inoculum; forest = young pine forest inoculum; control = non-mycorrhizal control.

The average total biomass per pot of the four associate plant individuals of *F*. *rubra* in mixtures was about 5–10 times larger than biomass of both focal species (Table 3A and 3B). Focal plants grown in mixtures with *F*. *rubra* produced less biomass than focal plants grown alone (RIIc < 0; Table 3C; Fig 1, Table C in S2 File). Inoculation with AM fungi led to an increase of focal plant biomass in mixtures with *F*. *rubra* compared to non-inoculated mixtures (Table 3A, Tables C and D in S2 File). The opposite pattern occurred for the biomass of *F*. *rubra*, whose biomass decreased in response to inoculation (Table 3B, Table C in S2 File), resulting in a greater benefit of inoculation to both focal species compared to the associate species (*F*. *rubra*) (dRIIi >0, Table 3D, Table D in S2 File).

**Table 3:** Results of linear models testing variation in different measures of plant response to competition. The measures were total biomass of **(a)** focal species (*L. hispidus*, *P. lanceolata*) and **(b)** associate species (*F. rubra*) when grown in mixture with each other; **(c)** total biomass growth response of focal species to competition (RIIc) and **(d)** the difference in growth response to inoculation of focal species and *F. rubra* when grown in mixture with each other (dRIIi _*mixture*_). Growth response parameters were calculated based on total plant biomass (root + shoot biomass), see Tables C-G in S2 File for results for root and shoot biomass separately. The explanatory factors tested were focal species (*L. hispidus*, *P. lanceolata*), inoculum origin (grassland, forest) and the interaction of both factors. For RIIc, values >0 indicate an increase and values <0 a decrease of plant biomass in response to competition. For, dRIIi _*mixture*_ values >0 indicate a larger and values <0 a smaller growth benefit from inoculation to focal species compared with *F. rubra*, when plants were grown in mixture with each other. Where factor levels differ at p<0.1, group means (*±* SE) are displayed, with different letters indicating significant differences (p<0.05) according to post-hoc tests (Tukey HSD).

types of plant growth response (total biomass, g)	explanatory factor	estimate	SE	DF	p-value	group means	
a) *Biomass _focal, mixture_	**mean value**	**0.8**	**0.1**	**0**	**<0.001**		
	**focal species**			**1**	**0.001**	*L*. *hispidus*^a^ = 0.6±0.2	
						*P*. *lanceolata*^b^ = 1.0±0.2	
	**inoculum origin**			**2**	**<0.001**	grassland^a^ = 1.5±0.2	
						forest^b^ = 0.9±0.2	
						control^c^ = 0.03±0.01	
	focal species x inoculum origin			2	0.472		
b) Biomass _associate, mixture_	**mean value**	**6.3**	**0.5**	**0**	**<0.001**		
	focal species			1	0.296		
	**inoculum origin**			**2**	**<0.001**	grassland^a^ = 4.5±0.3	
						forest^a^ = 5.1±0.4	
						control^b^ = 9.1±1.1	
	focal species x inoculum origin			2	0.210		
c) RIIc _focal_	**mean value**	**-0.67**	**0.03**	**0**	**<0.001**		
	focal species			1	0.280		
	**Inoculum origin**			**2**	**0.001**	grassland^a^ = -0.56±0.04	
						forest^b^ = -0.69±0.04	
						control^b^ = -0.77±0.05	
	**focal species x** **inoculum origin**	** **	** **	**2**	**0.001**	*L*. *hispidus*	*P*. *lanceolata*
						grassland^a^	grassland^a^
						= -0.59±0.07	= -0.52±0.04
						forest^a^	forest^a^
						= -0.74±0.06	= -0.65±0.06
						control^a^	control^b^
						= -0.61±0.06	= -0.93±0.01
d) dRIIi _mixture_	**mean value**	**1.16**	**0.03**	**0**	**<0.001**		
	**focal species**			**1**	**0.015**	*L*. *hispidus*^a^ = 1.20±0.03	
						*P*. *lanceolata*^b^ = 1.31±0.04	
	inoculum origin			1	0.062	grassland^a^ = 1.29±0.03	
						forest^a^ = 1.21±0.04	
	focal species x inoculum origin	** **	** **	**1**	0.075	*L*. *hispidus* grassland^a^ 1.20±0.04 forest^a^ 1.20±0.05	*P*. *lanceolata* grassland^a^ 1.39±0.02 forest^b^ 1.23±0.06

*statistical significance between factor levels calculated from log-transformed growth response parameters

### Effects of inoculum origin on competitive response

AM fungal inoculation increased focal plant biomass for plants grown alone or in competition with *F*. *rubra*. In mixtures with *F*. *rubra*, the grassland inoculum induced a larger increase in total and root biomass of focal species compared to the forest inoculum (Fig 1, Table 3A and 3C, Table E in S2 File). Both inocula led to the same magnitude of increase in focal shoot biomass (Table B in S2 File). The opposite pattern occurred for inoculation effects to *F*. *rubra* biomass. Focal biomass was lowest in the non-mycorrhizal control soils, higher in the forest inoculum and highest in the grassland inoculum, whilst *F*. *rubra* produced most biomass in the non-mycorrhizal control soils and least biomass in the grassland inoculum (Table 3A and 3B). This trend was the same for both focal species and all types of biomass (root, shoot, total biomass (Tables E and F in S2 File). In line with these patterns, the grassland inoculum induced a larger growth benefit of the focal species over the associate species (*F*. *rubra*) than the forest inoculum, when focal and associate plants were grown in mixtures (Table 3D, Table G in S2 File). However, these differences in dRIIi were marginally non-significant, due to smaller differential in the biomass response of *F*. *rubra* to grassland and forest inoculum (Table 3B and 3D).

### Combined effect of host species identity and inoculum origin on plant growth responses to competition

**Fig 2:**
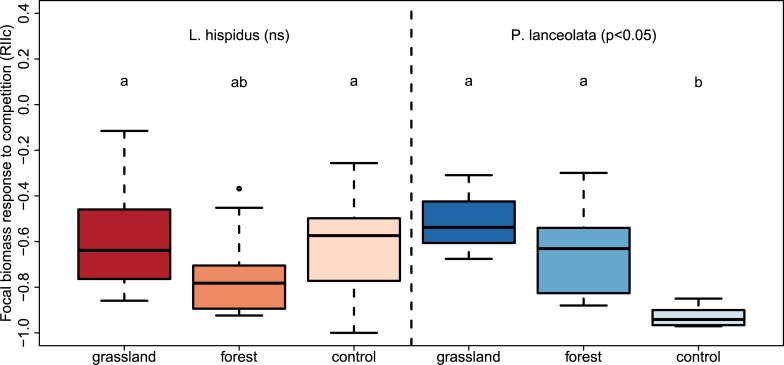
Competitive response of different focal species exposed to different inocula. Competitive response of both focal species (*L. hispidus, P. lanceolata*) was measured as index RII (RIIc, Eq (1)) based on plant biomass. Different letters indicate significant differences according to post hoc tests (p<0.05). Black lines indicate median values, boxes enclose quartiles, whiskers enclose 95% confidence intervals, and points indicate outlier values. grassland = grassland inoculum; forest = young pine forest inoculum; control = non-mycorrhizal control.

Effects of inoculation on competitive response differed between focal species (Table 3C). Inoculation did affect the competitive response of *P*. *lanceolata*, but not *L*. *hispidus*. In mixtures with *F*. *rubra*, *P*. *lanceolata* biomass was significantly larger when grown with AM fungal inoculum compared to non-mycorrhizal conditions (Table 3C, Fig 2, Table C in S2 File). Both focal species showed a tendency to produce more biomass in mixtures with *F*. *rubra*, when grown in grassland compared with forest inoculum, but there was high variation in growth response values and this difference was not statistically significant when analysing focal species separately (Table 3C, Fig 2, Table C in S2 File).

**Fig 3:**
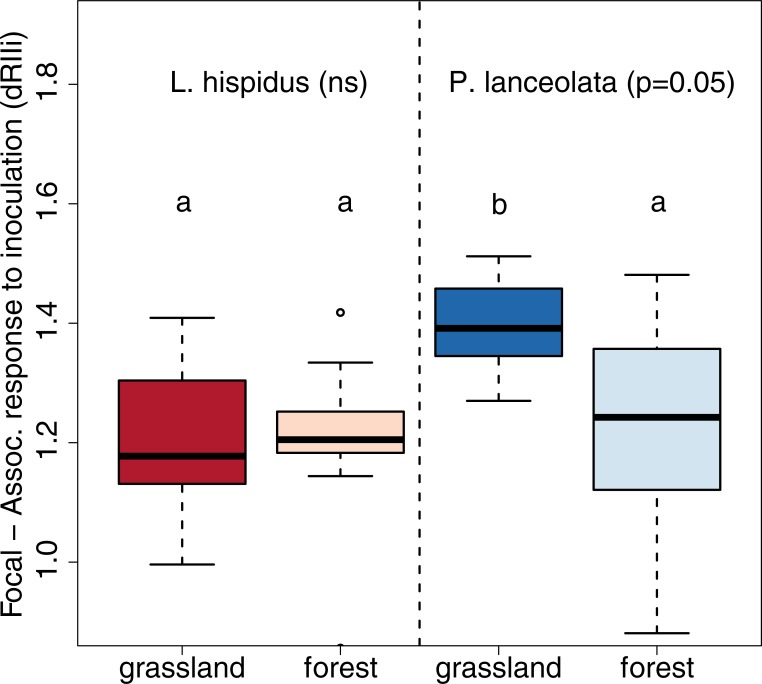
Differences in biomass response to inoculation for focal and associate species. Differences in biomass response to inoculation were measured based on the index dRII (Eq (2)) for comparing inoculation response of both focal species (*P. lanceolata*, *L. hispidus*) to the response of the associate species (*F. rubra*). Estimates are based on total plant biomass when plants were grown in competition with each other. Values >0 mean a greater increase and values <0 a smaller increase in focal biomass to inoculation compared to the growth response of *F. rubra* to inoculation. Different letters indicate significant differences according to post hoc tests (p<0.05). Black lines indicate median values, boxes enclose quartiles, whiskers enclose 95% confidence intervals, and points indicate outlier values. grassland = grassland inoculum; forest = young pine forest inoculum.

Differences between different combinations of focal species and inoculum origin were evident when comparing inoculation effects on focal and associate species, when both were grown together (dRIIi). Inoculation benefited the growth of focal plants more than the growth of *F*. *rubra* (dRIIi>0, Table 3D) when both specie were grown together. This benefit was larger for *P*. *lanceolata* compared to *L*. *hispidus* (Table 3D), with patterns being evident in total and shoot, but not root biomass (Table 3D, Table G in S2 File). Inoculation benefits to plant growth of *L*. *hispidus* compared to that of *F*. *rubra* were of the same magnitude for both inocula, while plant growth benefits to *P*. *lanceolata* were significantly larger for the grassland compared to the forest inoculum (Fig 3, Table 3D). This pattern was evident in total and root biomass, but not in shoot biomass Table G in S2 File).

## Discussion

The effects of AM fungi on plant diversity is a developing area of plant-mycorrhizal research [9, 15]. One proposed mechanism through which AM fungi might affect plant diversity and community structure is via their impact on plant-plant competition [4]. AM fungi may promote plant coexistence and thus plant diversity by balancing competition between plants, i.e. preventing competitive exclusion of the competitively weaker species through increasing their biomass relatively more compared to potentially stronger competitors [7, 19–22]. The results of our experiment support this idea. In line with our first hypothesis, we found that the presence of AM fungi reduces competition pressure from a dominant grass on a subordinate forb species, by increasing forb biomass, but decreasing grass biomass, thus balancing competition. Our results also demonstrate that while there is a positive net balancing effect of AM fungi, the magnitude of changes depends on the identity of both plant and AM fungal partners. In agreement with our second hypothesis, the grassland inoculum was generally more effective than the forest inoculum in increasing competitive response of the focal forb species. However, supporting our third hypothesis, the strength of this effect varied according to the habitat preference of the forb species, with a greater competitive response to the grassland inoculum observed in the grassland-preferring *P*. *lanceolata* than the forest-preferring *L*. *hispidus*. Thus, our findings provide evidence that natural AM fungal communities influence plant response to competition, but that the effects vary with AM fungal composition as well as the host plant species involved. This suggests that factors such as differential host plant–AM fungal relationships alongside co-adaptation between plant hosts and AM-fungal communities to local habitat conditions, might determine the impact of AM fungi on plant-plant interactions and ultimately plant community structure.

Since we used whole soil inocula, our results cannot be exclusively attributed to the different AM fungal communities in the inocula. Although we balanced the soil micorbial community as best as possible by applying a microbial filtrate of equally mixed forest and grassland soils to the all treatments, we cannot entirely exclude that the observed inoculation effects on plant biomass were not solely caused by differences in AM fungal communities. Nevertheless, the high AM fungal colonization of focal plant roots grown in inoculated soils suggests that the AM fungi play major role in this study system like in other studies using a similar approach [33–35, 54].

### Inoculation strengthens competitive response of the competitively weaker species

Our experiment showed that inoculation with AM fungi balanced plant competition by reducing competition pressure from the dominant grass and increasing the competitive response of the subordinate forb. Similar results have been reported earlier for a range of species combination [7, 19, 21, 23]. Yet, the small selection of host plant and AM fungal species used in the majority of previous experiments limits results-based predictions of AM fungal effects on plant competition in nature, cf. [7]. Conditions were more natural in our experiment, using whole AM fungal communities and mycorrhizal plant species that co-occur naturally in grasslands. Thus, the observation of balanced competition by AM fungi under these conditions provides good support for the notion that AM fungi mediate plant competition, thereby influencing plant coexistence and community structure in nature [9, 21, 55].

### Inoculation effects vary with inoculum and the host plant identity

Inoculation effects on competitive response of both focal species varied with the AM fungal inoculum used, supporting the idea that not only the presence of AM fungi, but also AM fungal abundance and composition have an effect on plant coexistence in natural ecosystems [4, 7, 22]. Indeed, AM fungal inocula differed in AM fungal abundance, with higher AM fungal biomass per unit soil in the grassland compared to the forest inoculum [36], although both inocula led to the similar degree of root colonization in our study. This suggests that differences in inoculum effectiveness were caused by `fine scale´ factors (sensu [4]), such as differences in the composition and diversity of AM fungi between inocula, with the grassland inoculum exhibiting higher diversity [36]. The high effectivity of the grassland inoculum may thus have been a result of it containing a higher number and abundance of beneficial AM fungi [15]. Additionally, diverse AM fungal communities may be more likely to contain particularly beneficial AM fungi [18, 22], and exhibit higher functional complementarity [56], which may also have contributed to the higher effectivity of the grassland inoculum. Since the colonization percentage of AM fungi in the roots of focal plants did not differ between the grassland and forest inocula, results suggest that differences in growth response to AM fungi tested in this study were due to differences in inoculum quality rather than quantity.

However, variation in inoculation effects was dependent on the combination of focal plant and AM fungal inoculum, suggesting that, besides AM fungal abundance and taxon composition, co-adaptation between host plants and local AM fungal communities might influence plant responses to inoculation [40, 55]. This indicates that the effectiveness of the grassland inoculum to increase competitive response of *P*. *lanceolata* may not only result from the AM fungal composition, but also from optimization of the symbiosis through adaptation by the plant species [41]. The high light requirement of *P*. *lanceolata* has likely created a strong affinity to open calcareous grasslands, the origin of the grassland inoculum, and the strong nutrient limitation of this habitat type might have triggered plant adaptation to specific AM fungal communities to optimize AM fungal mediated nutrient uptake [40, 55]. By contrast, the more flexible light requirement of *L*. *hispidus* paired with higher nutrient availability in the young pine forest might not have fostered strong co-adaptation between plants and AM fungal communities in young pine forest.

## Conclusion

Our findings indicate that the influence of AM fungi on plant coexistence in nature differs across habitats depending on the composition of the local AM fungal community. In this context, the high effectiveness of the grassland inoculum in improving the response of two competitively weaker forb species to competition with a dominant grass suggests that the diverse AM fungal communities in calcareous grasslands may be one factor promoting the high plant species diversity–especially of forbs–that is typical of this habitat. The clear positive effect of grassland inoculum on the grassland-preferring forb *P*. *lanceolata* further suggests that plant-plant interactions and consequently plant community structure is strongly interlinked with the local AM fungal community [57]. This has implication for nature conservation, as restoration of grasslands may benefit from coupling reintroduction of plant and AM fungal communities from target communities during restoration, cf. [57, 58]. This may be especially relevant in degraded or fragmented grasslands where altered soil conditions hamper the reestablishment target AM fungal communities or fragmentation and overgrowth limits the dispersal of AM fungal spores between suitable patches.

## Supporting information

S1 File Data tables used for analysis of biomass response to competition and inoculation (Tables A-C).
(PDF)

S2 File Fig A and Tables A-G.
(PDF)
