# Supplementary material for: Arbuscular mycorrhizal fungal community composition determines the competitive response of two grassland forbs
Source: PLoS One. 2019 Jul 10;14(7):e0219527. doi: 10.1371/journal.pone.0219527 (PMC6620016; doi:10.1371/journal.pone.0219527)
Supplement: S1 File — Data tables used for analysis of biomass response to competition and inoculation (Tables A-C). (PDF) [file pone.0219527.s001.pdf]

Table A. Data on focal species biomass and associate species biomass response to inoculation treatments.

pot #	focal species	inoculum	focal plants grown singly			focal plants grown in competition with *F. rubra*			*F. rubra* grown with focal species		
			shoot biomass (g)	root biomass (g)	total biomass (g)	shoot biomass (g)	root biomass (g)	total biomass (g)	shoot biomass (g)	root biomass (g)	total biomass (g)
1	*L. hispidus*	grassland	1.67	3.20	4.86	0.37	0.65	1.01	1.77	5.86	7.64
2	*L. hispidus*	grassland	0.02	0.02	0.04	0.62	1.07	1.69	1.48	1.98	3.46
3	*L. hispidus*	grassland	1.20	2.57	3.77	0.18	0.16	0.35	1.59	2.11	3.70
4	*L. hispidus*	grassland	1.40	6.37	7.77	0.88	2.74	3.62	2.83	2.20	5.03
5	*L. hispidus*	grassland	1.54	4.05	5.60	0.40	0.33	0.73	1.39	3.78	5.17
6	*L. hispidus*	grassland	0.61	0.40	1.01	0.54	1.87	2.41	1.39	1.85	3.24
7	*L. hispidus*	grassland	1.48	5.41	6.88	0.29	0.71	1.00	2.30	3.14	5.44
8	*L. hispidus*	grassland	1.12	4.59	5.72	0.16	0.36	0.51	1.96	3.15	5.10
9	*L. hispidus*	grassland	1.28	3.11	4.39	0.26	0.35	0.61	1.91	3.76	5.66
10	*L. hispidus*	grassland	1.13	4.42	5.55	0.43	0.58	1.01	1.94	4.51	6.45
11	*L. hispidus*	forest	1.42	2.68	4.10	0.19	0.16	0.35	2.37	2.45	4.82
12	*L. hispidus*	forest	0.50	1.09	1.59	0.31	0.94	1.24	1.76	3.51	5.27
13	*L. hispidus*	forest	1.11	1.84	2.95	0.42	0.15	0.57	2.04	2.33	4.37
14	*L. hispidus*	forest	0.91	1.77	2.68	0.19	0.27	0.46	1.57	1.34	2.91
15	*L. hispidus*	forest	1.11	2.61	3.72	0.34	0.22	0.56	1.46	3.32	4.78
16	*L. hispidus*	forest	1.02	1.40	2.42	0.06	0.07	0.13	1.87	1.95	3.82
17	*L. hispidus*	forest	0.78	0.76	1.54	1.19	0.33	1.52	1.97	3.78	5.75
18	*L. hispidus*	forest	1.28	2.45	3.72	0.10	0.09	0.18	1.88	6.65	8.53
19	*L. hispidus*	forest	0.93	1.73	2.66	0.07	0.09	0.16	1.69	1.26	2.95
20	*L. hispidus*	forest	1.54	5.94	7.48	0.11	0.14	0.25	2.10	2.27	4.37
21	*L. hispidus*	sterile	0.02	0.02	0.03	0.00	0.00	0.00	0.00	0.00	0.00
22	*L. hispidus*	sterile	0.06	0.02	0.08	0.00	0.01	0.01	2.96	5.88	8.84
23	*L. hispidus*	sterile	0.00	0.00	0.00	0.00	0.00	0.01	3.16	3.26	6.43
24	*L. hispidus*	sterile	0.02	0.02	0.04	0.01	0.01	0.02	3.30	2.93	6.22
25	*L. hispidus*	sterile	0.01	0.02	0.04	0.00	0.01	0.01	2.92	7.35	10.27
26	*L. hispidus*	sterile	0.01	0.02	0.02	0.00	0.00	0.00	2.96	4.02	6.98
27	*L. hispidus*	sterile	0.02	0.03	0.06	0.01	0.01	0.01	2.17	3.12	5.29
28	*L. hispidus*	sterile	0.01	0.04	0.05	0.01	0.01	0.01	2.61	15.11	17.72
29	*L. hispidus*	sterile	0.02	0.04	0.06	0.01	0.02	0.02	3.18	3.99	7.16
30	*L. hispidus*	sterile	0.00	0.01	0.01	0.00	0.01	0.01	3.06	6.87	9.92
31	*P. lanceolata*	grassland	2.76	5.14	7.90	0.64	0.71	1.35	1.80	2.03	3.82
32	*P. lanceolata*	grassland	1.87	3.07	4.94	0.56	0.76	1.31	2.32	2.92	5.24
33	*P. lanceolata*	grassland	1.97	2.97	4.94	0.61	1.05	1.66	1.47	2.72	4.19
34	*P. lanceolata*	grassland	2.15	3.25	5.40	1.50	1.36	2.86	0.91	4.19	5.10
35	*P. lanceolata*	grassland	2.05	2.75	4.80	0.62	0.71	1.33	2.02	2.39	4.41
36	*P. lanceolata*	grassland	1.67	1.63	3.30	0.58	0.47	1.05	1.80	2.29	4.09
37	*P. lanceolata*	grassland	2.97	3.61	6.58	1.03	0.58	1.61	1.21	2.18	3.40
38	*P. lanceolata*	grassland	2.75	3.26	6.01	0.90	1.29	2.20	1.54	1.50	3.03
39	*P. lanceolata*	grassland	2.53	2.23	4.76	1.11	0.80	1.91	1.30	2.50	3.80
40	*P. lanceolata*	grassland	2.46	3.16	5.62	0.76	1.63	2.39	1.36	2.06	3.42
41	*P. lanceolata*	forest	2.65	3.58	6.23	0.25	0.26	0.51	2.17	2.41	4.58
42	*P. lanceolata*	forest	1.67	2.82	4.48	0.46	0.88	1.34	1.64	5.59	7.23
43	*P. lanceolata*	forest	2.47	2.82	5.28	0.57	0.39	0.96	1.74	2.33	4.07
44	*P. lanceolata*	forest	1.69	2.92	4.61	0.78	0.86	1.64	1.07	2.13	3.21
45	*P. lanceolata*	forest	2.70	3.32	6.02	0.64	0.75	1.39	1.79	2.02	3.80
46	*P. lanceolata*	forest	2.09	2.23	4.32	0.71	0.90	1.60	1.71	3.99	5.69
47	*P. lanceolata*	forest	2.39	4.37	6.76	0.82	0.27	1.10	1.62	6.41	8.03
48	*P. lanceolata*	forest	2.18	3.01	5.19	0.23	0.17	0.40	2.01	3.06	5.07
49	*P. lanceolata*	forest	2.73	3.35	6.08	1.48	1.42	2.90	1.66	3.06	4.71
50	*P. lanceolata*	forest	2.06	2.66	4.71	0.21	0.13	0.34	2.02	6.64	8.66
51	*P. lanceolata*	sterile	0.22	0.27	0.49	0.01	0.02	0.02	3.03	4.28	7.30
52	*P. lanceolata*	sterile	0.88	1.06	1.94	0.01	0.01	0.02	3.40	4.46	7.86
53	*P. lanceolata*	sterile	0.19	0.22	0.41	0.04	0.03	0.07	2.65	12.21	14.86
54	*P. lanceolata*	sterile	0.30	0.27	0.57	0.01	0.01	0.02	3.07	4.98	8.05
55	*P. lanceolata*	sterile	0.96	2.04	2.99	0.01	0.02	0.03	2.79	10.92	13.71
56	*P. lanceolata*	sterile	0.41	0.40	0.80	0.01	0.03	0.03	2.38	4.99	7.37
57	*P. lanceolata*	sterile	0.64	0.86	1.50	0.01	0.01	0.02	2.38	6.80	9.18
58	*P. lanceolata*	sterile	0.30	0.24	0.54	0.03	0.03	0.06	3.48	18.72	22.21
59	*P. lanceolata*	sterile	0.23	0.17	0.39	0.01	0.03	0.04	2.76	2.89	5.65
60	*P. lanceolata*	sterile	0.69	0.65	1.34	0.04	0.05	0.09	3.32	4.40	7.72

Table B. Data on focal and associate species response to competition and inoculation treatment.

Data on growth response of focal species to competition (RIIcomp), and focal species and *F. rubra* inoculation (RII_{inoc}), as well as the difference in inoculation response to focal species and *F. rubra* (dRII), analysed in the present study.

pot #	focal species	inoculum	focal growth response to competition (RII_{comp}, Armas et al. 2004)			focal growth response to inoculation (RII_{inoc}, Armas et al. 2004)			*F. rubra* growth response to inoculation (RII_{inoc}, Armas et al. 2004)			difference in growth response of focal plants and *F. rubra* to inoculation (dRII)		
			RII_shoot	RII_root	RII_total	RII_shoot	RII_root	RII_total	RII_shoot	RII_root	RII_total	dRII_shoot	dRII_root	dRII_total
1	*L. hispidus*	grassland	-0.52	-0.68	-0.64	0.98	0.98	0.98	-0.20	0.06	-0.02	1.17	0.93	1.00
2	*L. hispidus*	grassland	-0.29	-0.52	-0.46	0.99	0.99	0.99	-0.28	-0.45	-0.39	1.27	1.44	1.38
3	*L. hispidus*	grassland	-0.72	-0.91	-0.86	0.96	0.93	0.94	-0.25	-0.43	-0.36	1.20	1.35	1.30
4	*L. hispidus*	grassland	-0.13	-0.11	-0.12	0.99	1.00	0.99	0.04	-0.41	-0.22	0.95	1.41	1.22
5	*L. hispidus*	grassland	-0.48	-0.83	-0.72	0.98	0.96	0.97	-0.31	-0.16	-0.21	1.29	1.13	1.18
6	*L. hispidus*	grassland	-0.36	-0.29	-0.31	0.99	0.99	0.99	-0.31	-0.48	-0.42	1.30	1.47	1.41
7	*L. hispidus*	grassland	-0.60	-0.66	-0.64	0.97	0.98	0.98	-0.07	-0.25	-0.18	1.04	1.24	1.16
8	*L. hispidus*	grassland	-0.76	-0.81	-0.80	0.95	0.97	0.96	-0.15	-0.25	-0.21	1.10	1.22	1.18
9	*L. hispidus*	grassland	-0.63	-0.81	-0.76	0.97	0.97	0.97	-0.16	-0.17	-0.16	1.13	1.13	1.13
10	*L. hispidus*	grassland	-0.45	-0.71	-0.64	0.98	0.98	0.98	-0.15	-0.08	-0.10	1.13	1.06	1.08
11	*L. hispidus*	forest	-0.70	-0.87	-0.81	0.96	0.93	0.94	-0.05	-0.36	-0.24	1.01	1.29	1.18
12	*L. hispidus*	forest	-0.55	-0.41	-0.45	0.98	0.99	0.98	-0.20	-0.20	-0.20	1.17	1.19	1.18
13	*L. hispidus*	forest	-0.43	-0.88	-0.71	0.98	0.92	0.97	-0.13	-0.39	-0.29	1.11	1.31	1.25
14	*L. hispidus*	forest	-0.69	-0.78	-0.75	0.96	0.96	0.96	-0.25	-0.59	-0.46	1.21	1.55	1.42
15	*L. hispidus*	forest	-0.52	-0.82	-0.71	0.98	0.95	0.97	-0.29	-0.23	-0.25	1.27	1.17	1.21
16	*L. hispidus*	forest	-0.89	-0.94	-0.92	0.88	0.83	0.86	-0.17	-0.46	-0.35	1.05	1.29	1.20
17	*L. hispidus*	forest	0.06	-0.74	-0.37	0.99	0.96	0.99	-0.15	-0.16	-0.16	1.14	1.13	1.14
18	*L. hispidus*	forest	-0.83	-0.93	-0.89	0.92	0.86	0.90	-0.17	0.12	0.04	1.09	0.75	0.86
19	*L. hispidus*	forest	-0.88	-0.92	-0.91	0.89	0.87	0.88	-0.22	-0.61	-0.46	1.11	1.48	1.33
20	*L. hispidus*	forest	-0.81	-0.88	-0.86	0.93	0.91	0.92	-0.11	-0.40	-0.29	1.04	1.31	1.21
21	*L. hispidus*	sterile	-1.00	-1.00	-1.00	NA	NA	NA	NA	NA	NA	NA	NA	NA
22	*L. hispidus*	sterile	-0.70	-0.62	-0.66	NA	NA	NA	NA	NA	NA	NA	NA	NA
23	*L. hispidus*	sterile	-0.89	-0.69	-0.77	NA	NA	NA	NA	NA	NA	NA	NA	NA
24	*L. hispidus*	sterile	-0.42	-0.37	-0.39	NA	NA	NA	NA	NA	NA	NA	NA	NA
25	*L. hispidus*	sterile	-0.70	-0.46	-0.56	NA	NA	NA	NA	NA	NA	NA	NA	NA
26	*L. hispidus*	sterile	-0.89	-0.83	-0.86	NA	NA	NA	NA	NA	NA	NA	NA	NA
27	*L. hispidus*	sterile	-0.55	-0.62	-0.59	NA	NA	NA	NA	NA	NA	NA	NA	NA
28	*L. hispidus*	sterile	-0.42	-0.56	-0.50	NA	NA	NA	NA	NA	NA	NA	NA	NA
29	*L. hispidus*	sterile	-0.37	-0.18	-0.26	NA	NA	NA	NA	NA	NA	NA	NA	NA
30	*L. hispidus*	sterile	-0.62	-0.51	-0.56	NA	NA	NA	NA	NA	NA	NA	NA	NA
31	*P. lanceolata*	grassland	-0.57	-0.63	-0.60	0.95	0.94	0.94	-0.24	-0.57	-0.46	1.19	1.51	1.41
32	*P. lanceolata*	grassland	-0.61	-0.61	-0.61	0.94	0.94	0.94	-0.12	-0.44	-0.33	1.06	1.38	1.27
33	*P. lanceolata*	grassland	-0.58	-0.50	-0.53	0.95	0.96	0.95	-0.33	-0.47	-0.43	1.28	1.42	1.38
34	*P. lanceolata*	grassland	-0.22	-0.39	-0.31	0.98	0.97	0.97	-0.53	-0.28	-0.34	1.50	1.25	1.31
35	*P. lanceolata*	grassland	-0.58	-0.63	-0.61	0.95	0.94	0.94	-0.18	-0.52	-0.40	1.13	1.45	1.35
36	*P. lanceolata*	grassland	-0.60	-0.74	-0.68	0.94	0.90	0.93	-0.24	-0.53	-0.44	1.18	1.44	1.36
37	*P. lanceolata*	grassland	-0.39	-0.69	-0.54	0.97	0.92	0.95	-0.41	-0.55	-0.51	1.38	1.47	1.46
38	*P. lanceolata*	grassland	-0.44	-0.41	-0.42	0.96	0.96	0.96	-0.31	-0.67	-0.55	1.28	1.63	1.51
39	*P. lanceolata*	grassland	-0.35	-0.59	-0.48	0.97	0.94	0.96	-0.38	-0.50	-0.46	1.36	1.44	1.42
40	*P. lanceolata*	grassland	-0.51	-0.31	-0.39	0.96	0.97	0.97	-0.37	-0.57	-0.51	1.32	1.54	1.47
41	*P. lanceolata*	forest	-0.80	-0.84	-0.83	0.88	0.83	0.86	-0.15	-0.51	-0.39	1.02	1.35	1.24
42	*P. lanceolata*	forest	-0.67	-0.56	-0.60	0.93	0.95	0.94	-0.28	-0.14	-0.18	1.21	1.09	1.12
43	*P. lanceolata*	forest	-0.60	-0.78	-0.70	0.94	0.89	0.92	-0.25	-0.52	-0.44	1.20	1.41	1.36
44	*P. lanceolata*	forest	-0.49	-0.57	-0.53	0.96	0.95	0.95	-0.46	-0.56	-0.53	1.42	1.50	1.48
45	*P. lanceolata*	forest	-0.56	-0.61	-0.59	0.95	0.94	0.94	-0.24	-0.58	-0.46	1.19	1.51	1.41
46	*P. lanceolata*	forest	-0.52	-0.55	-0.54	0.95	0.95	0.95	-0.26	-0.30	-0.29	1.22	1.25	1.24
47	*P. lanceolata*	forest	-0.47	-0.84	-0.66	0.96	0.84	0.93	-0.29	-0.08	-0.13	1.25	0.92	1.06
48	*P. lanceolata*	forest	-0.82	-0.90	-0.86	0.87	0.76	0.82	-0.19	-0.42	-0.34	1.05	1.18	1.16
49	*P. lanceolata*	forest	-0.21	-0.37	-0.30	0.98	0.97	0.97	-0.28	-0.42	-0.38	1.26	1.39	1.35
50	*P. lanceolata*	forest	-0.83	-0.92	-0.88	NA	NA	NA	-0.18	-0.06	-0.09	1.04	0.75	0.88
51	*P. lanceolata*	sterile	-0.98	-0.94	-0.96	NA	NA	NA	NA	NA	NA	NA	NA	NA
52	*P. lanceolata*	sterile	-0.97	-0.96	-0.97	NA	NA	NA	NA	NA	NA	NA	NA	NA
53	*P. lanceolata*	sterile	-0.84	-0.91	-0.88	NA	NA	NA	NA	NA	NA	NA	NA	NA
54	*P. lanceolata*	sterile	-0.98	-0.97	-0.97	NA	NA	NA	NA	NA	NA	NA	NA	NA
55	*P. lanceolata*	sterile	-0.94	-0.94	-0.94	NA	NA	NA	NA	NA	NA	NA	NA	NA
56	*P. lanceolata*	sterile	-0.96	-0.92	-0.94	NA	NA	NA	NA	NA	NA	NA	NA	NA
57	*P. lanceolata*	sterile	-0.98	-0.96	-0.97	NA	NA	NA	NA	NA	NA	NA	NA	NA
58	*P. lanceolata*	sterile	-0.89	-0.91	-0.90	NA	NA	NA	NA	NA	NA	NA	NA	NA
59	*P. lanceolata*	sterile	-0.96	-0.90	-0.93	NA	NA	NA	NA	NA	NA	NA	NA	NA
60	*P. lanceolata*	sterile	-0.86	-0.84	-0.85	NA	NA	NA	NA	NA	NA	NA	NA	NA

Table C. Dataset on plant root colonization in resonse to competition and inoculation.

focal species	inoculum	focal plants grown singly				focal plants grown in competition with *F. rubra*				colonization response to comptition (RII_{comp}; Armas et al., 2004)			
		Hyphae (%)	Arbuscules (%)	Vesicles (%)	Coils (%)	Hyphae (%)	Arbuscules (%)	Vesicles (%)	Coils (%)	RII_Hyphae	RII_Arbuscules	RII_Vesicles	RII_Coils
L. hispidus	grassland	86.7	44.2	0.8	1.7	90.0	62.5	2.5	2.5	0.01	0.06	-0.30	-0.27
L. hispidus	grassland	82.5	62.5	0.8	10.8	81.7	41.7	0.0	5.0	-0.04	-0.15	-1.00	0.07
L. hispidus	grassland	95.0	70.0	0.0	5.0	90.8	60.8	0.0	0.8	0.01	0.04	-1.00	-0.68
L. hispidus	grassland	94.2	51.7	3.3	0.8	93.3	37.5	0.0	0.8	0.03	-0.20	-1.00	-0.68
L. hispidus	grassland	84.2	51.7	18.3	3.3	91.7	45.8	3.3	0.8	0.02	-0.10	-0.17	-0.68
L. hispidus	forest	84.2	41.7	0.0	0.0	96.7	64.2	5.8	3.3	0.04	0.05	0.71	0.54
L. hispidus	forest	89.2	46.7	3.3	0.8	81.7	54.2	0.0	3.3	-0.04	-0.04	-1.00	0.54
L. hispidus	forest	78.3	56.7	0.0	1.7	96.7	47.5	0.8	0.0	0.04	-0.10	-0.09	-1.00
L. hispidus	forest	95.8	79.2	0.0	1.7	90.0	47.5	12.5	0.0	0.01	-0.10	0.85	-1.00
L. hispidus	forest	96.7	67.5	1.7	0.8	94.2	36.7	10.0	1.7	0.03	-0.23	0.82	0.25
P. lanceolata	grassland	57.5	14.2	11.7	1.7	72.5	19.2	10.0	0.0	0.08	0.29	0.40	-1.00
P. lanceolata	grassland	44.2	10.0	0.0	0.8	76.7	25.0	1.7	0.0	0.10	0.40	-0.44	-1.00
P. lanceolata	grassland	72.5	5.8	0.8	0.8	90.8	50.8	3.3	0.0	0.19	0.65	-0.13	-1.00
P. lanceolata	grassland	93.3	18.3	9.2	0.0	51.7	0.8	1.7	0.8	-0.09	-0.86	-0.44	0.00
P. lanceolata	grassland	44.2	5.0	0.0	0.8	75.0	15.0	2.5	1.7	0.09	0.17	-0.27	0.33
P. lanceolata	forest	51.7	3.3	6.7	0.0	80.0	41.7	10.8	4.2	0.02	0.20	0.33	0.92
P. lanceolata	forest	76.7	12.5	4.2	0.0	81.7	36.7	9.2	0.8	0.03	0.14	0.25	0.67
P. lanceolata	forest	67.5	38.3	6.7	0.8	92.5	53.3	11.7	0.0	0.09	0.31	0.36	-1.00
P. lanceolata	forest	99.2	54.2	10.0	0.0	85.0	17.5	1.7	0.0	0.05	-0.23	-0.54	-1.00
P. lanceolata	forest	91.7	30.8	0.0	0.0	83.3	19.2	6.7	1.7	0.04	-0.18	0.10	0.82
