# Supplementary material for: Arbuscular mycorrhizal fungal community composition determines the competitive response of two grassland forbs
Source: PLoS One. 2019 Jul 10;14(7):e0219527. doi: 10.1371/journal.pone.0219527 (PMC6620016; doi:10.1371/journal.pone.0219527)
Supplement: S2 File — Fig A and Tables A-G. (PDF) [file pone.0219527.s002.pdf]

Supplementary Material (S2 File)

Arbuscular mycorrhizal fungal community composition determines the competitive response of two grassland forbs

Authors:

Lena Neuenkamp[1,2], Martin Zobel[1], Eva Lind[1], Maret Gerz[1], Mari Moora[1]

Affiliation:

[1] Institute of Ecology and Earth Sciences, University of Tartu, Lai 40, 51005 Tartu, Estonia

[2] Institute of Plant Sciences, University of Bern, Altenbergrain 21, 3013 Bern, Switzerland

Corresponding author:

Lena Neuenkamp (lena.neuenkamp@ips.unibe.ch, twitter: @LNeuenkamp)

Number of supplementary items:

1 figure, 10 tables

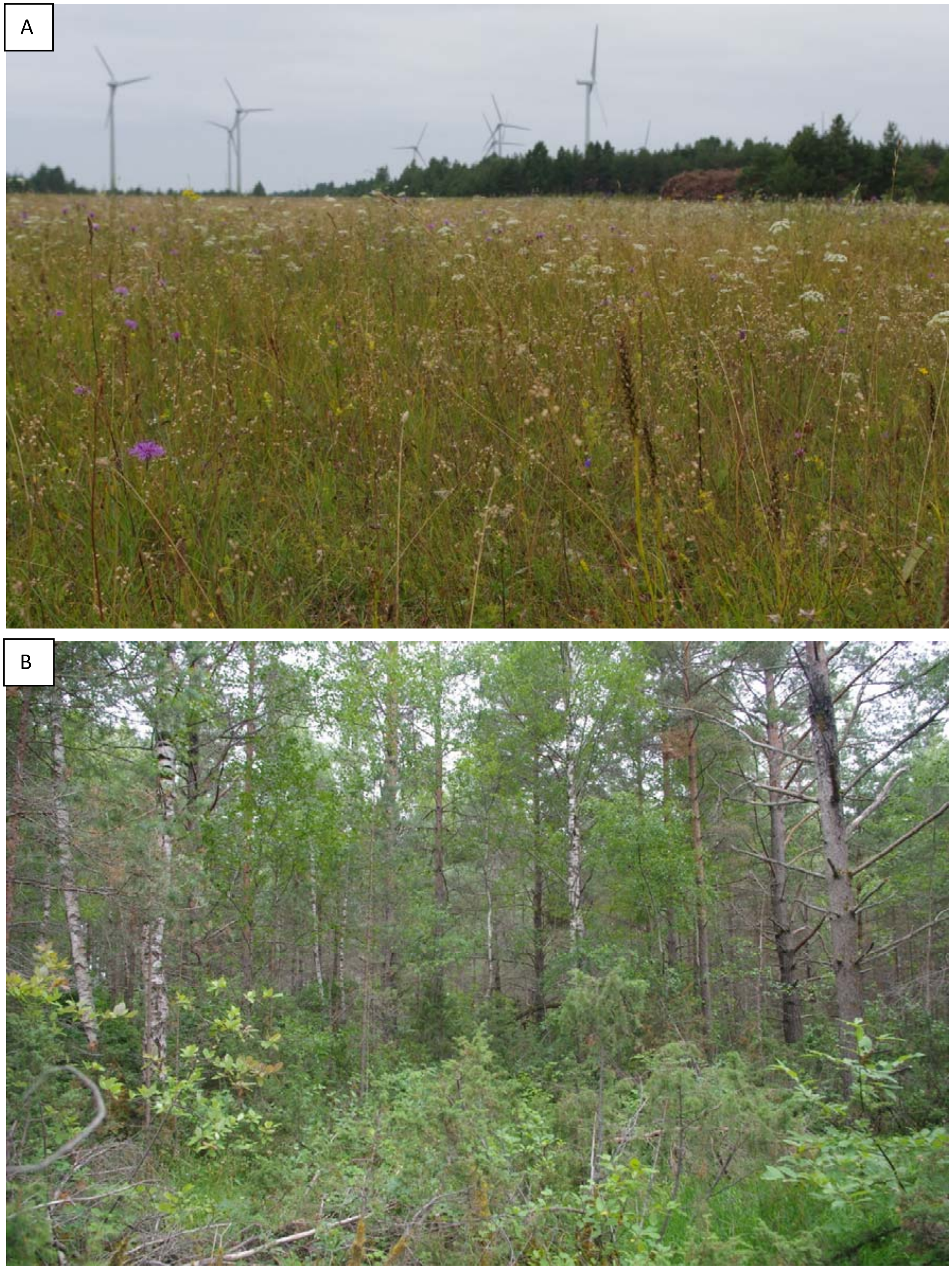

Fig A. Photographs of the two successional stages of calcareous grasslands sampled in this study.
(A) open grassland and **(B)** young pine forest. These successional stages form a sequence of regeneration succession covering 0-60 years after cessation of management (grazing/mowing/tree removal). The composition of plant and AM fungal communities in these sites has been studied in an earlier study by Neuenkamp et al. (2018).

Table A. Results of linear models assessing variation in root colonization for plants grown in monoculture. Variation in root colonization (%) is tested for plants growing in monoculture and in response to focal plant species (*L. hispidus*, *P. lanceolata*), inoculum origin (grassland inoculum, forest inoculum) and their interaction. Where factor levels differ at $p<0.1$, group means (± SE) are displayed, with different letters indicating significant differences ($p<0.05$) according to post-hoc tests (Tukey HSD).

AM fungal structures	explanatory factor	estimate	SE	DF	p-value	group means
hyphal colonization	**mean value**	**79.3**	**3.9**	**0**	**<0.001**	*L. hispidus* = 88.7±2.0<br>*P. lanceolata* = 69.9±6.5
	**focal species**			**1**	**0.012**	
	inoculum origin			1	0.267	
	focal species x inoculum origin			1	0.287	
vesicular colonization	**mean value**	**3.9**	**1.1**	**0**	**0.003**	
	focal species			1	0.378	
	inoculum origin			1	0.604	
	focal species x inoculum origin			1	0.315	
arbuscular colonization	**mean value**	**38.2**	**5.4**	**0**	**<0.001**	*L. hispidus* = 57.2±3.9<br>*P. lanceolata* = 19.2±5.3
	**focal species**			**1**	**<0.001**	
	inoculum origin			1	0.138	
	focal species x inoculum origin			1	0.253	
coil colonization	**mean value**	**1.6**	**0.6**	**0**	**0.011**	*L. hispidus* =2.7±1.0<br>*P. lanceolata* = 0.5±0.2<br>grassland =2.6±1.0<br>forest = 0.6±0.2
	**focal species**			**1**	**0.003**	
	**inoculum origin**			**1**	**0.045**	
	focal species x inoculum origin			1	0.165	

Table B. Results of linear models assessing variation in the response of root colonization to competition. Linear models assessed variation in the relative response of root colonization (%) of focal plants to competition (RIIc) by different AM fungal structures in relation to focal species (*L. hispidus*, *P. lanceolata*), inoculum origin (grassland inoculum, forest inoculum) and their interaction. Mean RIIc values > 0 indicate an increase and mean RIIc values < 0 a decrease in root colonization of the focal species in response to competition with *F. rubra*. Where factor levels differ at p<0.1, group means (± SE) are displayed, with different letters indicating significant differences (p<0.05) according to post-hoc tests (Tukey HSD).

AM fungal structures	explanatory factor	estimate	SE	DF	p-value	group means
hyphal colonization	**mean value**	**0.03**	**0.01**	**0**	**0.018**	
	focal species			1	0.083	*L. hispidus*[a] =0.01±0.01<br>*P. lanceolata*[b] = 0.06±0.02
	inoculum origin			1	0.723	
	focal species x inoculum origin			1	0.454	
vesical colonization	mean value	-0.13	0.14	0	0.352	
	focal species			1	0.452	
	**inoculum origin**			**1**	**0.018**	grassland[a] =-0.44±0.14<br>forest[b] = 0.18±0.19
	focal species x inoculum origin			1	0.166	
arbuscular colonization	mean value	0.01	0.07	0	0.931	
	focal species			1	0.267	
	inoculum origin			2	0.740	
	focal species x inoculum origin			1	0.815	
coil colonization	mean value	-0.26	0.16	0	0.125	
	focal species			1	0.847	
	inoculum origin			1	0.178	
	focal species x inoculum origin			1	0.650	

Table C. Results of linear models assessing variation in plant growth response to competition. Linear models assessed variation in plant growth responses (shoot biomass, root biomass) to competition (RIIc) in relation to focal species (*L. hispidus*, *P. lanceolata*), inoculum origin (grassland inoculum, forest inoculum, sterile inoculum) and their interaction. Mean RIIc values >0 indicate an increase and mean RIIc values <0 a decrease in plant biomass in response to competition. Where factor levels differ at p<0.1, group means (± SE) are displayed, with different letters indicating significant differences (p<0.05) according to post-hoc tests (Tukey HSD).Different letters indicate statistical differences among group means (p<0.05).

Type of plant biomass (g)	explanatory factor	estimate	SE	DF	p-value	group means	
shoot biomass	**mean value**	**-0.63**	**0.03**	**0**	**<0.001**		
	focal species			1	0.121		
	**inoculum origin**			**2**	**<0.001**	grassland[a] = -0.49±0.04<br>forest[a]= -0.61±0.05<br>sterile[b] = -0.80±0.05	
	**focal species x inoculum origin**			**2**	**0.026**	*L. hispidus*<br>grassland[a] = -0.49±0.06<br>forest[a]= -0.63±0.09<br>sterile[a] = -0.66±0.07	*P. lanceolata*<br>grassland[a] = -0.48±0.04<br>forest[a]= -0.60±0.06<br>sterile[b] = -0.93±0.02
root biomass	**mean value**	**-0.70**	**0.03**	**0**	**<0.001**		
	focal species			1	0.345		
	**inoculum origin**			**2**	**0.007**	grassland[a] = -0.59±0.05<br>forest[b]= -0.76±0.04<br>sterile[b] = -0.76±0.05	
	**focal species x inoculum origin**			**2**	**<0.001**	*L. hispidus*<br>grassland[a] = -0.63±0.08<br>forest[a]= -0.82±0.05<br>sterile[a] = -0.58±0.07	*P. lanceolata*<br>grassland[a] = -0.55±0.04<br>forest[a]= -0.69±0.06<br>sterile[b] = -0.93±0.01

Table D. Results of linear models assessing variation in focal plant biomass grown in monoculture. Linear models assessed variation in focal plant biomass (total biomass, shoot biomass, root biomass) when grown without *F. rubra* in relation to the factors focal species (*L. hispidus*, *P. lanceolata*), inoculum origin (grassland inoculum, forest inoculum, non-mycorrhizal control) and their interaction. Where factor levels differ at p<0.1, group means (± SE) are displayed, with different letters indicating significant differences (p<0.05) according to post-hoc tests (Tukey HSD).

Type of plant biomass (g)	explanatory factor	estimate	SE	DF	p-value	group means	
total biomass	**mean value**	**3.3**	**0.3**	**0**	**<0.001**		
	**focal species**			**1**	**0.001**	*L. hispidus*[a] = 2.6±0.5<br>*P. lanceolata*[b] = 4.0±0.4	
	**inoculum origin**			**2**	**<0.001**	grassland[a] = 5.0±0.4<br>forest[a]= 4.3±0.4<br>control[b] = 0.6±0.2	
	focal species x inoculum origin			2	0.343		
shoot biomass	**mean value**	**1.2**	**0.1**	**0**	**<0.001**		
	**focal species**			**1**	**<0.001**	L. hispidus[a] = 0.7±0.1<br>P. lanceolata[b] = 1.7±0.2	
	**inoculum origin**			**2**	**<0.001**	grassland[a] =1.7±0.2<br>forest[a]= 1.7±0.2<br>control[b] = 0.2±0.1	
	**focal species x inoculum origin**			**2**	**0.002**	*L. hispidus*<br>grassland[a] =1.2±0.2<br>forest[a]= 1.1±0.1<br>control[b] = 0.0±0.0	*P. lanceolata*<br>grassland[a] =2.3±1.3<br>forest[a]= 2.2±0.1<br>control[b] = 0.5±0.1
root biomass	**mean value**	**2.1**	**0.2**	**0**	**<0.001**		
	**focal species**			**1**	**<0.001**	L. hispidus[a] = 1.9±0.4<br>P. lanceolata[b] = 2.3±0.3	
	**inoculum origin**			**2**	**<0.001**	grassland[a] = 3.3±0.3<br>forest[a]= 2.7±0.3<br>control[b] = 0.3±0.1	
	**focal species x inoculum origin**			**2**	**<0.001**	*L. hispidus*<br>grassland[a] = 3.4±0.6<br>forest[a]= 2.2±0.5<br>control[b] = 0.02±0.00	*P. lanceolata*<br>grassland[a] = 3.1±0.3<br>forest[a]= 3.1±0.2<br>control[b] = 0.6±0.2

Table E. Results of linear models assessing variation in focal plant biomass grown in competition. Linear models assessed variation in focal plant biomass (shoot biomass, root biomass) when grown in mixture with *F. rubra* in relation to the factors focal species (*L. hispidus*, *P. lanceolata*), inoculum origin (grassland inoculum, forest inoculum, non-mycorrhizal control) and their interaction. Where factor levels differ at p<0.1, group means (± SE) are displayed, with different letters indicating significant differences (p<0.05) according to post-hoc tests (Tukey HSD).Different letters indicate statistical differences among group means (p<0.05).

Type of plant biomass (g)	explanatory factor	estimate	SE	DF	p-value	group means
*shoot biomass	**mean value**	**0.4**	**0.1**	**0**	**<0.001**	
	**focal species**			1	**<0.001**	*L. hispidus*[a] = 0.2±0.1<br>*P. lanceolata*[b] = 0.5±0.1
	**inoculum origin**			**2**	**<0.001**	grassland[a] = 0.6±0.1<br>forest[a]= 0.5±0.01<br>control[b] = 0.01±0.00
	focal species x inoculum origin			2	0.575	
*root biomass	**mean value**	**0.4**	**0.1**	**0**	**<0.001**	
	**focal species**			**1**	**<0.001**	*L. hispidus*[a] = 0.4±0.1<br>*P. lanceolata*[b] = 0.5±0.1
	**inoculum origin**			**2**	**<0.001**	grassland[a] = 0.9±0.1<br>forest[b]=0.4±0.1<br>control[c] = 0.01±0.00
	focal species x inoculum origin			2	0.287	

*statistical significance between factor levels calculated from log-transformed growth response parameters

Table F. Results of linear models assessing variation in the biomass of the associate species grown with focal plants.

Linear models assessed variation in associate (*F. rubra*) plant biomass (shoot biomass, root biomass) when grown in mixture with the focal species (*L. hispidus, P. lanceolata*) in response to the factors focal species (*L. hispidus*, *P. lanceolata*), inoculum origin (grassland inoculum, forest inoculum, non-mycorrhizal control) and their interaction. Where factor levels differ at $p<0.1$, group means (± SE) are displayed, with different letters indicating significant differences ($p<0.05$) according to post-hoc tests (Tukey HSD).Different letters indicate statistical differences among group means ($p<0.05$).

Type of plant biomass (g)	explanatory factor	estimate	SE	DF	p-value	group means
shoot biomass	**mean value**	**2.1**	**0.1**	**0**	**<0.001**	
	focal species			1	0.775	
	**inoculum origin**			**2**	**<0.001**	grassland[a] = 1.7±0.1<br>forest[a]= 1.8±0.1<br>control[b] = 2.8±0.2
	focal species x inoculum origin			2	0.207	
*root biomass	**mean value**	**4.2**	**0.4**	**0**	**<0.001**	
	focal species			1	**<0.001**	*L. hispidus*[a] = 3.8±0.5<br>*P. lanceolata*[b] = 4.6±0.7
	**inoculum origin**			**2**	**<0.001**	grassland[a] = 2.9±0.2<br>forest[a]= 3.3±0. 4<br>control[b] = 6.4±1.0
	focal species x inoculum origin			2	0.288	

*statistical significance between factor levels calculated from log-transformed growth response parameters

Table G. Results of linear models assessing variation of in biomass differences between focal species and associate species.

Linear models assessed variation in the difference between focal (*L. hispidus*, *P. lanceolata*) and associate plant (*F. rubra*) growth responses to inoculation when grown in mixture with each other ($dRIIi_{mixture}$). Mean $dRIIi_{mixture}$ values >0 indicate a larger and mean $dRIIi_{mixture}$ values <0 a smaller growth benefit from inoculation to focal species compared to *F. rubra,* when both are grown in mixture with each other. Variation is assessed in the response of shoot and root biomass, and in relation to the factors focal species (*L. hispidus*, *P. lanceolata*), inoculum origin (grassland inoculum, forest inoculum) and their interaction.).Where factor levels differ at p<0.1, group means (± SE) are displayed, with different letters indicating significant differences (p<0.05) according to post-hoc tests (Tukey HSD).Different letters indicate statistical differences among group means (p<0.05).

Type of plant biomass (g)	explanatory factor	estimate	SE	DF	p-value	group means	
shoot biomass	**mean value**	**1.83**	**0.02**	**0**	**<0.001**		
	**focal species**			**1**	**0.019**	*L. hispidus*^{a} = 1.14±0.02<br>*P. lanceolata*^{b} = 1.23±0.03	
	inoculum origin			1	0.098	grassland^{a} = 1.34±0.04<br>forest^{a}= 1.24±0.05	
	focal species x inoculum origin			1	0.538		
root biomass	**mean value**	**1.29**	**0.03**	**0**	**<0.001**		
	focal species			1	0.107		
	inoculum origin			1	0.103		
	focal species x inoculum origin			1	0.077	*L. hispidus*<br>grassland^{a} = 1.24±0.06<br>forest^{a} = 1.25±0.07	*P. lanceolata*<br>grassland^{a} = 1.45±0.03<br>forest^{a*} =1.29±0.07
